# Long-term sexual functioning in germ-cell tumor survivors

**DOI:** 10.1186/s12885-020-07301-6

**Published:** 2020-08-20

**Authors:** M. Chovanec, L. Vasilkova, L. Petrikova, J. Obertova, P. Palacka, K. Rejlekova, Z. Sycova-Mila, K. Kalavska, D. Svetlovska, B. Mladosievicova, J. Mardiak, M. Mego

**Affiliations:** 1 2nd Department of Oncology, Faculty of Medicine, Comenius University, Klenova 1, Bratislava, 833 10 Slovak Republic; 2 2nd Department of Oncology, Faculty of Medicine, Translational Research Unit, Comenius University, Bratislava, Slovak Republic; 3 Department of Medical Oncology, National Cancer Institute, Bratislava, Slovak Republic; 4 Department of Psychology, Faculty of Philosophy, Comenius University, Bratislava, Slovak Republic; 5 Institute of Pathological Physiology, Faculty of Medicine, Comenius University, Bratislava, Slovak Republic; 6 Department of Oncohematology I, Oncohematology Clinic, National Cancer Institute, Bratislava, Slovak Republic

**Keywords:** Germ cell tumors, Long-term toxicity, Sexual impairment, Chemotherapy, Radiotherapy

## Abstract

**Background:**

Survivors of germ-cell tumors (GCT) may suffer from long-term adverse consequences. Our study was conducted to assess a long-term sexual functioning in GCT survivors.

**Methods:**

GCT survivors (*N* = 170) from the National Cancer Institute in Slovakia completed a Sexual Function Questionnaire that was modified from PROMIS Sexual Function and Satisfaction Questionnaire 9-year median follow up (range 5–32) as a primary exploratory aim. Study groups consisted of 17 survivors (10%) who had active surveillance (AS, controls), and 153 (90%) survivors who received treatment beyond orchiectomy (Tx), including cisplatin-based chemotherapy (CT, *N* = 132; 78%), radiotherapy to the retroperitoneal lymph nodes (RT, *N* = 12; 7%) or both (CTRT, *N* = 9; 5%).

**Results:**

In univariate analysis, treatment of any type resulted in difficulty to maintain erection during sexual intercourse compared to patients treated with AS (*P* = 0.04). Survivors who received CTRT had lower ability to achieve orgasm during sexual activities (*P* = 0.04) and they reported disappointment with their overall quality of sex life (*P* = 0.002). The number of attempts to initiate sexual intercourse did not differ. Sexual relationships caused none or mild anxiety and the desire to be sexually active was higher after CTRT (*P* = 0.05). Multivariable analysis confirmed that orgasmic dysfunction after ≥400 mg/m^2^ of cisplatin and issues in maintaining erection after Tx were independent of retroperitoneal lymph-node dissection (*P =* 0.03 and *P =* 0.04, respectively). Survivors were disappointed with the quality of sex life and had stronger desire to be sexually active independent of age, (*P =* 0.01 and *P =* 0.05, respectively).

**Conclusions:**

This study identified an impairment in sexual function may represent an issue for long-term GCT survivors. Treatment with chemotherapy plus radiotherapy were associated with disappointment and stronger sexual desire, while a higher cumulative dose of cisplatin may be responsible for orgasmic dysfunction.

## Background

Testicular germ-cell tumors (GCTs) are an example of successfully integrated multidisciplinary curative treatments in oncology [1]. Cure for testicular GCTs entails a different need for treatment approach from orchiectomy only cured individuals to ones needing radiotherapy to retroperitoneal lymph nodes, adjuvant chemotherapy, one line of chemotherapy, post-chemotherapy surgery or salvage chemotherapy including high-dose regimens [2–7]. Each of these approaches bring the different burden of morbidity to GCT survivors. GCT survivors may achieve decades of additional life, and as such, they serve as an excellent model for studying the late toxicity of cancer treatment [1]. GCT survivors reportedly suffer from an array of long-term adverse sequelae of these treatments [8]. Sexual functioning is an important element contributing to the quality of life. Disturbances in sexual function were reported in breast cancer survivors previously [9, 10]. The psychosexual health was also reported to be a serious issue in young male adults cured from a malignancy [11]. Certain sexual dysfunctions were reported recently from a Danish nation-wide cohort of long-term GCT survivors including erectile and orgasmic problems in patients treated for GCTs [12]. The lives of GCT survivors may extend to several decades. These men are generally young adults at the time of their diagnosis, therefore their sex life is essential in terms of fathering children, establishing families, and overall quality of life. Our study aimed to independently assess the self-reported sexual issues in long-term GCT survivors and to investigate the impact of distinct treatment modalities and cumulative burden of given-treatments.

## Methods

### Patients

This prospective study was conducted as a part of an ongoing translational study (Protocol IZLO-1) evaluating long-term toxicities in GCT survivors. Herein, we assessed the long term sexual functioning in survivors cured from their GCT with chemotherapy and/or radiotherapy. The study included GCT survivors treated at the National Cancer Institute in Bratislava, Slovakia from 1983 to 2012 who subsequently underwent an annual long-term follow up at our outpatient clinic. Long-term annual follow up has been implemented at our institution per our internal guidelines for testicular cancer survivorship program. The study included GCT survivors more than 5 years from the last treatment. However, a significant number of patients who completed the treatment between 1983 and 2012 were lost to follow-up. We are unable to estimate this number and the reasons for non-adherence to the long-term follow-up. Therefore, the study population consisted of survivors adherent to our observational protocol.

Similarly as described previously [13], survivors were divided into subgroups according to different curative treatments received: orchiectomy alone (AS = active surveillance; control group); and treatment beyond orchiectomy: radiotherapy (RT), chemotherapy (CT), chemotherapy and radiotherapy (CTRT), any treatment (Tx). To evaluate the consequences of cumulative burden of cisplatin-based chemotherapy, groups of < 400 mg/m^2^ vs ≥ 400 mg/m^2^ of cisplatin and ≥ 400 mg/m2 cisplatin versus the control group were assessed. As the study is continuously accruing new survivors reaching the 5-year time interval after treatment, the number of patients increased from 155 to 170 in this study. Of 170 patients within the currently presented study, there were 123 overlappings with the previous one [13]. The reason there was not a 100% overlap is explained by the fact that some patients did not complete all questionnaires during one visit. Patients with metastatic non-seminoma who had enlarged residual lymph nodes > 1 cm in size after chemotherapy, underwent a retroperitoneal-lymph node dissection (RPLND) if rendered operable by a urologic surgeon (Table 1).

**Table 1:** Patients’ characteristics

	All (%)	Treatment group				
		AS	RT	CT	CTRT	***P*** value
	***N*** = 170 (100%) (10)(((100)					
**Age (years)**						
Median (range)	40 (21–77)					
**Follow up (years)**						
Median (range)	9 (5–32)					
**Histology**						
Pure seminoma	47 (28)	8	11	19	9	< 0.001
Non-seminoma/mixed GCT	116 (68)	9	1	106	0	
Histology unknown	7 (4)	0	0	7	0	
**Primary tumor**						
Gonadal	158 (92)	17	12	120	9	0.39
Primary retroperitoneal	9 (6)	0	0	9	0	
Primary mediastinal	3 (2)	0	0	3	0	
**IGCCCG risk group**						
Good risk	77 (49)	0	0	70	7	0.06
Intermediate risk	21 (12)	0	0	21	1	
Poor risk	29 (17)	0	0	29	0	
unknown	1 (0.5)	0	0	1	0	
**Initial stage**						
I	42 (26)	17	12	11	2	< 0.001
I.S-III.A	77 (45)	0	0	70	7	
III.B	21 (11)	0	0	21	0	
III.C	29 (18)	0	0	29	0	
Stage unknown		0	0	1	0	
**Treatment**						
AS	17 (10)					
RT only	12 (7)					
- adjuvant RT	12(7)					
CT only	132 (78)					
- 1st line only	112 (65)					
- more than 1st line	20(12)					
CTRT	9 (5)					
- 1st line treatment	8 (4.5)					
- 2nd line treatment	1 (0.5)^b^					
**Initial chemotherapy**						
BEPx3	43 (26)					
EPx4	22 (13)					
BEPx4	43 (25)					
other^a^	33 (19)					
**Post-chemotherapy RPLND**						
no	134	17	12	98	8	0.001
yes	35	0	0	34	1	
**Time from the end of treatment (years)**						
5–10	100 (59)	15	8	70	7	0.01
11–15	41 (24)	1	4	35	1	
> 15	29 (17)	1	0	27	1	

^a^Other regimens included: adjuvant BEPx2 (*N* = 10), BEPx2 for stage I.S disease (*N* = 1), TBEPx4 (*N* = 6), TBEPx2 + EPx2 (*N* = 1), VAB VAI (*N* = 1), VIPx4 (*N* = 1), VIPx6 (*N* = 1), VIP × 4 + PVB × 1 (*N* = 1), BEPx2 + EPx2 (*N* = 1), high dose VIP (*N* = 3), TBEPx4 + TEPx1 (*N* = 1), GETUG 13 experimental arm (*N* = 2), PVB × 4 (*N* = 2), carboplatin + cyclophosphamide × 6 (*N* = 1)

^b^patient who had a relapse in CTRT group was initially treated with RT to the retroperitoneum and experienced systemic relapse 8 months later. He was treated with carboplatin + cyclophosphamide × 6. Subsequent relapse was treated with second line VIPx6

Abbreviations: *IGCCCG* International Germ Cell Cancer Collaborative Group, *NA* Not available, *AS* Active surveillance, *RT* Radiotherapy, *CT* Chemotherapy, *CTRT* Chemotherapy and radiotherapy, *RPLND* Retroperitoneal lymph node dissection

Institutional Review Board (IRB) (Ethics committee of Slovak National Cancer Institute in Bratislava) approved the study and patients provided their consent in written form according to the protocol approved by IRB. Patients were enrolled between September 2015 and April 2017.

### Study measures

GCT survivors completed the questionnaire of sexual functioning during the follow-up visit performed annually. The questionnaire was modified from PROMIS Sexual Function and Satisfaction (SexFS) and hypogonadism symptom questionnaires and was filled at a median 9 years of follow up (range 5–32) as a primary exploratory aim. The questionnaire was filled in a secluded office in an environment under control and uniform for all participating survivors. Survivors completed the questionnaire undisturbed and alone in the office. The study nurse was available for assistance in case of the need at all times.

Data collected from all participants included age, histology of the tumor, disease characteristics, treatment administered, clinical examination, and sexual function questionnaires. All data were collected at the outpatient clinic and at the office of clinical trials during the visit. The PROMIS SexFS domain from the PROMIS questionnaire consisted of a range of specific questions assessing the self-reported sexual functioning including the sexual desire, erectile function, orgasmic function and were enriched with questions assessing the disappointment and anxiety resulting from sex life. The sections that were deemed not relevant for this study were not included from the PROMIS SexFS domain. The PROMIS SexFS is a widely used and validated questionnaire tool for self-reported sexual quality of life in patients with cancer [14]. Question items were slightly modified per regional and religious concerns where appropriate. Supplementary Table 1 presents the question items along with their question stems and response options and indicates which items were part of the original PROMIS SexFS domain as well as items that were subsequently included by our team of psychologists. Question items were scored according to Likert-scale**. A** higher score for questions 1–5 indicates a better sexual function. A higher score for questions 6–8 indicates a higher symptom burden (Supplementary Table 1). Peripheral blood was drawn on the same day when survivors completed the questionnaire. The testosterone level was analyzed by enzyme-linked immunosorbent assay from the collected samples to assess the differences in serum levels between the study groups.

### Statistical analysis

Characteristics of patients were categorized and ordered into table form. To compare the characteristics of treatment groups versus controls, t-tests were used for continuous variables. A non-parametric Mann-Whitney U test or Kruskal-Wallis test was used where appropriate (data distributed not normally) to analyze the associations between distinct sexual functions and delivered treatments. Within the multivariable analysis, we designed a general linear model to adjust for age and we used multivariate analysis of variance to adjust for retroperitoneal lymph-node dissection (RPLND).

The median of follow-up was defined as the median time of observation from the last treatment visit in all GCT survivors. All reported *P* values were two-sided. A *P* value < 0.05 was considered significant. Statistical software NCSS 10, 2015 (Hintze J, 2015, Kaysville, Utah, USA) was used to perform all statistical analyses.

## Results

### Patients’ characteristics

The survivor population in the study consisted of 170 GCT survivors with a median age of 40 years at the follow-up (range: 21–77 years). Patients’ characteristics are summarized in Table 1.

Data regarding the distribution of patients treated with radiotherapy to the retroperitoneal lymph nodes for stage I or stage II seminoma, subsequent chemotherapy for contralateral primary, and chemotherapy for relapse after radiotherapy were provided in our previous study [13]. When we assessed the age distribution we have found that GCT survivors treated with any treatment were older than ones who underwent active surveillance (median 40 vs 33 years, *P* < 0.01).

### The impact of treatment on sexual desire and activity

The level of overall sexual desire did not differ across the study groups regardless of treatment administered (all *P* > 0.05). Survivors across the study groups reported 3–6 attempts to be sexually active in the past 30 days. The attempted frequency of sexual activity did not differ across the study groups (*P* > 0.5) **(**Table 2**)**.

**Table 2:** Univariate analysis of self-reported long-term sexual functioning in testicular germ-cell tumor survivors^a^

		N	Mean	SEM	Median	***P*** value
**Sexual desire**	AS	17	3.29	0.22	3	NA
	RT	12	3.08	0.31	3	0.74
	CT	132	3.14	0.08	3	0.45
	CTRT	9	3.44	0.30	3	0.79
	Tx	153	3.15	0.08	3	0.51
**Attempts to initiate intercourse**	AS	17	2.41	0.35	2	NA
	RT	12	1.58	0.41	1.5	0.14
	CT	132	2.45	0.15	2	0.94
	CTRT	9	2.66	0.54	3	0.68
	Tx	153	2.40	0.14	2	0.95
**Achieve erection**	AS	17	4.47	0.39	5	NA
	RT	12	3.50	0.47	4.5	0.12
	CT	132	3.94	0.15	5	0.24
	CTRT	9	2.89	0.55	3	0.05
	Tx	153	3.84	0.14	5	0.18
**Maintain erection**	AS	17	4.47	0.38	5	NA
	RT	12	3.33	0.45	4	0.06
	CT	132	3.77	0.14	4	0.07
	CTRT	9	3.00	0.53	4	0.04
	Tx	153	3.69	0.14	4	0.04
**Achieve orgasm**	AS	17	4.64	0.28	5	NA
	RT	12	4.33	0.34	5	0.59
	CT	130	4.12	0.13	5	0.08
	CTRT	9	3.22	0.49	4	0.03
	Tx	151	4.09	0.12	5	0.08
**Disappointed with quality of sex life**	AS	17	1.35	0.21	1	NA
	RT	12	2.08	0.25	2	0.06
	CT	130	1.61	0.07	1	0.33
	CTRT	9	2.56	0.26	3	< 0.01
	Tx	151	1.70	0.07	1	0.18
**Anxiety from sexual relationships**	AS	17	1.41	0.22	1	NA
	RT	12	1.83	0.27	1	0.37
	CT	130	1.38	0.07	1	0.64
	CTRT	9	1.56	0.26	1	0.74
	Tx	151	1.43	0.07	1	0.81
**Strong desire to be sexually active**	AS	17	2.88	0.24	3	NA
	RT	12	3.08	0.29	3	0.71
	CT	130	2.80	0.09	3	0.74
	CTRT	9	3.67	0.36	4	0.05
	Tx	151	2.87	0.09	3	0.94

^a^in comparison with patients treated with orchiectomy and active surveillance (group AS)

Abbreviations: *AS* Active surveillance (controls), *RT* Radiotherapy, *CT* Chemotherapy, *CTRT* Chemotherapy and radiotherapy, *Tx* any treatment, *NA* Not applicable, *SEM* Standard error of the mean

### The impact of treatment on long-term erectile function

Survivors treated with CTRT reported a lower frequency of achieved erections compared to the active surveillance controls. While the controls reported being able to have an erection in the most attempted cases, survivors treated with CTRT could achieve an erection in less than 50% of attempts (*P* = 0.05) (Table 2). During the sexual intercourse, survivors reported difficulty maintaining erection when treated with any type of treatment compared to controls (*P* = 0.04). The most pronounced difficulty was seen in the CTRT group (*P* = 0.04**) (**Table 2), where survivors reported that keeping erection was hard during intercourse. Controls reported being able to maintain an erection in most attempted cases (Table 2). Furthermore, survivors treated with chemotherapy with **≥**400 mg/m^2^ of cisplatin (4 or more cycles of chemotherapy) had a non-significant trend to reduced ability to maintain erection compared to AS controls (*P* = 0.06) (Table 3)**.**

**Table 3:** Subgroup univariate analysis of sexual functioning in GCT survivors treated with orchiectomy only versus with chemotherapy containing ≥400 mg/m^2^ of cisplatin or < 400 mg/m^2^ of cisplatin^a^

		N	Mean	SEM	Median	***P*** value
**Sexual desire**	AS	17	3.29	0.21	3	NA
	< 400	51	3.31	0.14	3	0.94
	≥400	80	3,05	0.09	3	0.23
		**N**	**Mean**	**SEM**	**Median**	***P*** **value**
**Attempts to initiate intercourse**	AS	17	2.41	0.39	2	NA
	< 400	51	2.61	0.25	3	0.65
	≥400	80	2.38	0.18	2	0.91
		**N**	**Mean**	**SEM**	**Median**	***P*** **value**
**Achieve erection**	AS	17	4.47	0.42	5	NA
	< 400	51	4.12	0.22	5	0.55
	≥400	80	3.90	0.19	5	0.17
		**N**	**Mean**	**SEM**	**Median**	***P*** **value**
**Maintain erection**	AS	17	4.47	0.41	5	NA
	< 400	51	3.84	0.23	5	0.16
	≥400	80	3.78	0.18	4	0.06
		**N**	**Mean**	**SEM**	**Median**	***P*** **value**
**Achieve orgasm**	AS	17	4.65	0.38	5	NA
	< 400	50	4.34	018	5	0.37
	≥400	80	3.97	0.17	5	0.03
		**N**	**Mean**	**SEM**	**Median**	***P*** **value**
**Disappointed with quality of sex life**	AS	17	1.35	0.22	1	NA
	< 400	50	1.52	0.11	1	0.67
	≥400	79	1.65	0.10	1	0.24
		**N**	**Mean**	**SEM**	**Median**	***P*** **value**
**Anxiety from sexual relationships**	AS	17	1.41	0.21	1	NA
	< 400	50	1.24	0.09	1	0.23
	≥400	79	1.48	0.09	1	0.97
		**N**	**Mean**	**SEM**	**Median**	***P*** **value**
**Strong desire to be sexually active**	AS	17	2.88	0.26	3	NA
	< 400	50	2.88	0.15	3	0.98
	≥400	79	2.76	0.11	3	0.63

Abbreviations: *AS* Active surveillance, < 400 - chemotherapy containing < 400 mg/m2 of cisplatin,

≥400 - chemotherapy containing ≥400 mg/m2 of cisplatin, SEM – standard error of the mean, NA – not applicable

^a^one patient receiving VAB VAI regimen was excluded from this analysis

### The impact of treatment on long-term orgasmic function

If sexually active, survivors treated with chemotherapy reported non-significantly decreased ability to achieve orgasm compared to controls (*P* = 0.08). Treatment with CTRT was associated with a significantly reduced ability to have an orgasm (approximately 50% of cases) compared to controls (*P* = 0.03**) (**Table 2**)**. Survivors treated with **≥**400 mg/m^2^ of cisplatin during their course of treatment had a lower ability to achieve orgasm compared to controls (*P* = 0.03) while being able to have an orgasm in more than 50% of attempts (Table 3).

### Psychosocial issues resulting from long-term sexual impairment

Curative treatment for GCT was not associated with anxiety from sexual relationships in any group of our GCT survivors (all *P* > 0.05). However, survivors treated with CTRT expressed to be disappointed with their sex life compared to controls (*P* = 0.003) and they had a stronger desire to be sexually active (*P* = 0.05). A non-significant trend to disappointment with sex life was seen in the RT group (*P* = 0.06) (Table 2).

### The impact of testosterone levels on sexual functioning

GCT Survivors may often suffer from symptoms resulting from low testosterone levels [15]. We have measured testosterone levels on a day of the clinical visit when patients completed the questionnaire. Reproducible results of testosterone levels were available from a total of 142 survivors. Upon analysis, there was no statistically significant difference between groups (all *P* > 0.05) (Table 4).

**Table 4:** Differences of levels of testosterone (ng/dL) in treatment groups of GCT survivors versus survivors who underwent active surveillance

Treatment group	N	Mean	SEM	Median	***P*** value
AS	16	762.1	103.5	705	NA
RT	9	682.4	138.0	537	0.57
CT	102	652.7	43.7	475	0.09
CTRT	5	576.8	150.0	566	0.36
Tx	116	651	40.5	492.5	0.10

Abbreviations: *AS* Active surveillance (controls), *RT* Radiotherapy, *CT* Chemotherapy, *CTRT* Chemotherapy and radiotherapy, *Tx* any treatment, *NA* Not applicable, *SEM* Standard error of the mean

### Multivariable analysis

We have performed a multivariable analysis in all instances where univariate analysis indicated significant differences. A multivariable analysis has shown that difficulty in maintaining erection was independent of RPLND in survivors with any treatment vs controls (*P =* 0.04). Adjusting for RPLND, survivors treated with ≥400 mg/m^2^ of cisplatin compared to AS had significantly more difficulties in achieving orgasm (*P =* 0.03). Adjusting for age in survivors treated with CTRT vs AS, statistical significance was lost in the ability to achieve an erection (*P* = 0.09), the ability to maintain an erection (*P* = 0.26), and in the ability to achieve an orgasm (*P* = 0.19). Disappointment with sex life and a strong desire to be sexually active remained higher in CTRT as opposed to AS (*P* = 0.01 and *P* = 0.05, respectively). Significance was also lost in the ability to maintain an erection in survivors treated with Tx versus AS (*P* = 0.19).

## Discussion

In this prospectively performed observational survivorship study, we have shown that self-reported sexual impairment may be a persistent problem years to decades after curative treatment for GCT. Our study comprehensively assessed the long-term sexual functioning considering the aspects of dysfunctions, distinct types of treatment, and cumulative burden of chemotherapy and chemotherapy plus radiotherapy. Because of the excellent curability of GCTs with a multi-modality approach, GCT survivors represent a unique model population to study late toxic sequelae of curative treatments.

A study by Joly et al. have found decreased sexual enjoyment, decreased desire, and infertility among GCT survivors [16]. Dahl et al. reported worse ejaculatory function and overall satisfaction with sexual relationships gradually increasing with the age of GCT survivors [17]. However, none of both studies assessed the erectile nor orgasmic function in this patient population. The largest study assessing the long-term sexual functioning to date from the Danish nationwide cohort of 2260 GCT survivors after 17 years of follow up was performed by Bandak et al. Authors used the IIEF-15 questionnaire, which covers erectile function, orgasmic function, sexual desire, and overall satisfaction. In this large cohort, authors found erectile dysfunction in patients treated with BEP (bleomycin, etoposide, cisplatin) and subsequent surgery, radiotherapy, or ones treated with more than one line of chemotherapy. Orgasmic dysfunction was found in all treatment groups excluding patients receiving BEP alone [12]. Consistently with these findings, we have found erectile dysfunction in men cured from GCT. However, men who were treated with radiotherapy only or chemotherapy only did not report significantly worse function than those on active surveillance. Higher burden of symptoms was seen in two scenarios: i) survivors treated with chemotherapy with **≥**400 mg/m^2^ of cisplatin for achieving orgasm; these are the patients treated with 4 cycles of chemotherapy for intermediate and poor-risk disease according to the International Germ Cell Cancer Collaborative Group (IGCCCG) criteria, or patients receiving salvage chemotherapy for relapsed disease, ii) survivors treated with both chemotherapy plus radiotherapy, who had erectile and orgasmic dysfunctions and were disappointed with their sex life. These patients had a stronger desire to be sexually active. While this group of patients seems to carry a certain level of frustration from sexual activity, overall satisfaction with sex life among the study groups was balanced in our study. According to the results of multivariable analysis, age was probably a confounding factor for erectile and orgasmic dysfunction, but not for disappointment with sex life and a strong desire to be sexually active for those treated with CTRT. The ability to maintain an erection in men receiving Tx or **≥** 400 mg/m^2^ of cisplatin was impaired independently of post-chemotherapy RPLND. A study from Denmark on 401 survivors, reported that survivors treated with chemotherapy and subsequent RPLND reported ejaculatory dysfunction, however, authors did not look at orgasmic function. Erectile dysfunction was present in 17.6% of survivors but was different among treatment groups [18]. A study by Eberhard et al. in 129 GCT survivors 3–5 years after treatment has reported lower sexual desire OR 6.7 (95% CI 2.1–21) and erectile dysfunction OR 3.8 (95% CI 1.4–10) compared to age-matched controls. No changes in premature or delayed ejaculation and sexual satisfaction, as well as no changes between the treatment groups, were reported [19]. Another study by Bandak et al. assessed long-term sexual function in men with bilateral testicular cancer but did not find further sexual impairment with treatment for contralateral testicular GCT [20].

Achieved results suggest, that correct risk stratification to avoid a common overtreatment in GCT patients is essential. It is imperative that treating clinician makes correct diagnosis to avoid overtreatment with four cycles of triple agent chemotherapy for good-risk disease or to avoid overtreatment of stage I disease with positive but declining tumor markers after orchiectomy. Further radiotherapy to post-chemotherapy residual tumor masses should be also avoided because the cumulative toxicity of this approach represents an increasing risk for cardiovascular diseases, secondary malignancies, cognitive functioning as well as some sexual impairment per our findings [13, 21, 22]. However, due to very small treatment groups, we need larger studies to validate these results. The underlying mechanisms for sexual impairment are not clear. Hypogonadism found in about 1/3 of GCT survivors has been linked to the risk of cardiovascular morbidity [15]. Our study did not show significant changes in testosterone levels across treatment groups. Erectile dysfunction may stem from underlying endothelial dysfunction which may be a common pathogenic factor for cardiovascular disease as well as erectile impairment [23]. The role of psychological distress from the diagnosis or presence of GCT may represent another risk factor for erectile dysfunction, as a study on 241 GCT survivors compared to 223 healthy controls have shown erectile dysfunction in 37.7% of patients before chemotherapy and persisting after treatment [24]. Orgasmic dysfunction may stem from several factors, including chemotherapy or radiotherapy-induced neuropathy, lower levels of testosterone or psychological issues, and possible use of selective serotonin reuptake inhibitors [25–28]. While retrograde ejaculation is the common adverse outcome of retroperitoneal lymph node dissection [29, 30], the orgasmic sensation is usually retained. However, some reports suggest, that orgasmic sensation may be reduced in patients with retrograde ejaculation [25]. However, the complex long-term effects of chemotherapy and radiotherapy to the retroperitoneum are unknown, and systemic long-acting mechanisms need to be explored in further translational studies.

Our study has certain strengths and limitations. The strengths entail prospective cohort of GCT survivors, which may be challenging to recruit due to low adherence to surveillance years after treatment. Another strength is a comprehensive assessment of distinct types of treatment modalities as well as the cumulative burden of chemotherapy and their effect on long-term sexual functioning. There is a number of limitations that need mentioning. Our study consisted of GCT survivors followed-up at our survivorship clinic upon completing their curative treatment. However, a number of survivors were lost from the long-term follow-up for unknown reasons. We are unable to find the cofounding factor for the non-adherence, which we consider a limitation of this study. Survivors adhering to long-term follow up may have a higher symptom burden, which creates a possible bias of the study. Among other limitations are smaller patient subgroups in some of the assessed categories, small control group, and using a modified PROMIS questionnaire with several non-validated questions. Patients subjected to active surveillance were younger and their follow up was shorter than of those who had treatment. Despite numerous limitations of our study, some findings from our questionnaire are consistent with the findings of Bandak et al. [12], therefore we may consider these independently acquired data with a different tool as a step towards validating the results.

## Conclusion

In conclusion, our results indicate, that perceived sexual impairment may be a persisting problem for long-term GCT survivors. Treatment with chemotherapy plus radiotherapy was associated with disappointment with sex life and a strong desire to be sexually active. A higher cumulative dose of cisplatin may be responsible for orgasmic dysfunction in GCT survivors.

Future research in this field is warranted to explore the underlying mechanisms of sexual dysfunctions and to refine the treatment strategies to minimize late adverse consequences on the sex life of GCT survivors.

## Supplementary information

**Additional file 1: Table S1.** Question stems and response options for evaluated items.

## Data Availability

The datasets and materials used and analyzed during the reported study are available at 2nd Department of Oncology, Faculty of Medicine, Comenius University, Bratislava, Slovakia. The datasets used and analyzed during the current study are available from the corresponding author in reasonable request.

## Abbreviations

AS: Active surveillance
CT: Chemotherapy
CTRT: Chemotherapy + radiotherapy
GCT: Germ cell tumor
Gy: Gray unit
IRB: Institutional Review Board
NA: Not applicable
RT: Radiotherapy
RPLND: Retroperitoneal lymph node dissection
SEM: Standard error of the mean
SexFS: Sexual Function and Satisfaction
