# Supplementary material for: Long-term sexual functioning in germ-cell tumor survivors
Source: BMC Cancer. 2020 Aug 20;20:779. doi: 10.1186/s12885-020-07301-6 (PMC7439516; doi:10.1186/s12885-020-07301-6)
Supplement: Supplementary file 1 — Additional file 1: Table S1. Question stems and response options for evaluated items. [file 12885_2020_7301_MOESM1_ESM.docx]

**Supplementary table 1. Question stems and response options for evaluated items**

| Question item ID when applicable (as appeared on PROMIS questionnaire) | Item stem  In the past 30 days: | Item categories |
| --- | --- | --- |
| SFINT102 | What was the level of your sexual desire? | 1 – very low/almost none  2 – low  3 – intermediate  4 – high  5 – very high |
| NA | How many times did you attempt to have sexual intercourse? | 0 – did not attempt  1 – 1-2 attempts  2 – 3-4 attempts  3 – 5-6 attempts  4 – 7-10 attempts  5 – more than 10 attempts |
| SFEFN004 | How would you describe the FREQUENCY of your erections during intercourse? | 0 – I had no attempts  1 – almost never/never  2- several times (less than 50% of times when having intercourse)  3 – sometimes (approximately 50% of times when having intercourse)  4 – most of the time (more than 50% of times when having intercourse)  5 – almost each time/each time |
| SFEFN001, SFEFN 103 | During intercourse – how difficult was it for you to maintain erection until the end of the intercourse? | 0 – I did not attempt intercourse  1 – extremely difficult  2 – very difficult  3 – difficult  4 – mild difficulty  5 – easy |
| NA | How often did you achieve an orgasm, If sexually active (with or without sexual intercourse) | 0 – I did not have sexual stimulation/intercourse  1 – almost never/never  2 – several times (less than 50% of times)  3 – sometimes (approximately 50% of times)  4 – most of the times (significantly more than 50% of times)  5 – almost always/always |
| SFSAT001 | Are you disappointed with the quality of your sex life? | 1 – no  2 – mildly disappointed  3 – disappointed  4 – very disappointed |
| NA | Did you feel a distress from sexual relations/activities? | 1 – no  2 – a little  3 – quite a lot  4 – very distressed |
| SFINT102 | Did you have a strong desire to be sexually active? | 1 – no  2 – a little  3 – quite strong  4 – very strong |
